# Cardiorespiratory Arrest in the Postoperative Period of Cardiovascular Surgery: What Changes?

**DOI:** 10.21470/1678-9741-2025-0020

**Published:** 2025-09-26

**Authors:** Hélio Penna Guimarães, Isadora Salvador Rocco, Walter José Gomes, Solange Guizilini

**Affiliations:** 1 Department of Surgery, Disciplina de Cirurgia Cardiovascular, Escola Paulista de Medicina, Universidade Federal de São Paulo - Unifesp, São Paulo, São Paulo, Brazil; 2 Postgraduation Program in Cardiology, Escola Paulista de Medicina, Universidade Federal de São Paulo - Unifesp, São Paulo, São Paulo, Brazil

**Figure:**
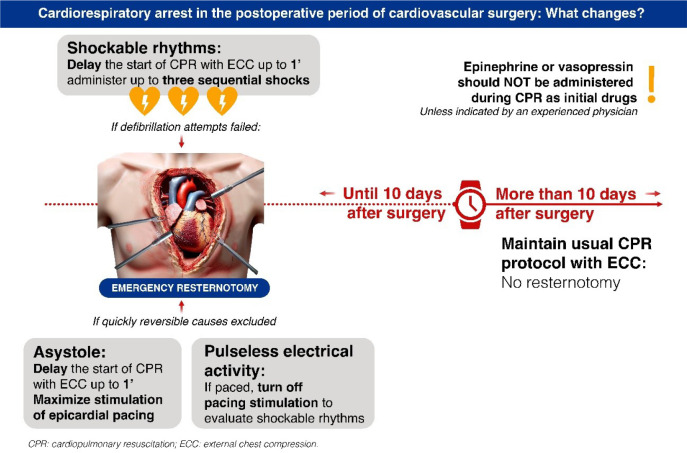

The occurrence of cardiorespiratory arrest (CRA) in cardiac surgery units is an uncommon but extremely severe event, involving various factors that can directly impact the recovery of spontaneous circulation and the post-CRA prognosis. Therefore, it demands specific approaches and effective protocols for its management.

Most CRAs after cardiac surgery have a reversible cause, such as hypovolemia or cardiac tamponade. In 60 - 70% of these cases, conventional cardiopulmonary resuscitation (CPR) is not effective, necessitating a resternotomy within five minutes of the arrest onset^[1,2]^. It is worth noting that this CRA often occurs in a highly monitored environment, facilitating immediate recognition and rapid and safe intervention by intensivist physicians and cardiovascular surgeons trained to perform advanced life support maneuvers^[1-3]^.

Among the most common causes of CRA after cardiac surgery, hypovolemia is prevalent despite a total body fluid increase of up to 30% after cardiopulmonary bypass (CPB). The depletion of intravascular volume is associated with capillary leakage, hemodilution, redistribution, and polyuria. Hemorrhage may be related to blood dyscrasia or surgical causes, including residual effects of heparin, platelet dysfunction (in number and activity), coagulation factor deficiencies, and extensive fibrinolysis, as well as bleeding from sternal wires and anastomotic or side branches of graft sites.

Low cardiac output may occur due to decreased ventricular function after CPB, myocardial edema, metabolic dysfunction, ischemia, reperfusion injury, and hypocalcemia, returning to baseline values within 24 to 72 hours. Right ventricular failure accounts for approximately 20% of low cardiac output states in the postoperative period. Graft and valve dysfunctions are serious complications, often masked in the immediate postoperative period by temporary epicardial pacing. Although the incidence of early graft dysfunction is low (3%), it can be fatal depending on the presentation, as well as valve dysfunction^[2,3]^. Cardiac tamponade can be challenging to diagnose because the "classic" signs may be challenging to unveil (*e.g.*, in hypovolemic and beta-blocked patients with left ventricular dysfunction)^[3]^.

It is crucial to recognize that postoperative CRA not only challenges the healthcare team in terms of immediate response but also emphasizes the importance of preventive strategies and early interventions during the perioperative period. Essentially, differences between CRA post-cardiac surgery and CRA that typically manifest in other clinical conditions should be considered.

A meta-analysis^[3]^ of 23 studies on CPR not associated with cardiac surgery showed that the incidence of pericardial injury after external chest compression (ECC) is 8.9%, the rate of sternal fracture is 15%, and the rate of rib fracture is 32%. Additionally, cases of myocardial lacerations, cardiac chamber ruptures, prosthetic valve dehiscence, vascular dissection, papillary muscle rupture, and 10% of conduction system injuries have been reported.

In this context, the Society of Thoracic Surgeons (STS)^[4]^, following the methodologies of the American College of Cardiology Foundation/American Heart Association (AHA), proposed recommendations for the management of CRA in the postoperative period of cardiovascular surgery, aiming to optimize care for patients experiencing such an adverse outcome. These recommendations address the need to distinguish between patients with less and more than 10 postoperative days to follow specific protocols, highlighting the importance of individualized care. This differentiation recognizes the potential variations in physiology and clinical conditions of patients at different postoperative stages, which may influence the most appropriate therapeutic strategies.

Thus, some of the recommended modifications to the traditional AHA algorithm should be applied in cases of CRA after cardiac surgery. These modifications include the recommendation for emergency resternotomy as a possible intervention of the resuscitation protocol up to 10 days after surgery; beyond 10 days postoperatively, the traditional protocol should be followed^[5]^.

The evidence supporting the performance of an initial cycle of ECC before defibrillation or the immediate implementation of epicardial pacing is unclear, as potential unnecessary harm should be considered^[6,7]^. The use of drugs in CRA after cardiac surgery does not provide conclusive evidence of benefit or harm. For example, epinephrine may cause severe hypertension and bleeding in patients who recover spontaneous circulation^[7,8]^.

Extracorporeal membrane oxygenation (ECMO) has emerged as a key tool in treating refractory cardiac arrest following cardiac surgery, particularly in younger patients, improving survival and neurological outcomes^[9]^. Its effectiveness relies on the rapid stabilization of hemodynamics and brain perfusion, making prompt initiation critical after identifying cardiac arrest. Despite its potential, ECMO is complex and requires a multidisciplinary team. Hemorrhage is the most common complication, affecting over 50% of cases due to the need for anticoagulation. To maximize ECMO’s benefits while minimizing risks, the development of specific protocols, specialized training, and resource allocation are essential for managing post-cardiac surgery resuscitation^[10-11]^.

In summary, the guidelines recommend specific changes to the usual CPR protocols for post-cardiac surgery cases up to 10 days after surgery^[5-8]^ (Table 1).

**Table 1:** Changes to the usual cardiopulmonary resuscitation (CPR) recommendations for post-cardiac surgery:

For shockable rhythms, delay the start of CPR with external chest compressions (ECC) by up to one minute to administer up to three sequential shocks.
Perform emergency resternotomy after three failed defibrillation attempts, initiating ECC until the establishment of direct intrathoracic compression.
In cases of asystole, the delay can be up to one minute to maximize stimulation with epicardial pacing.
In instances of pulseless electrical activity with an operational pacemaker, deactivate the pacemaker briefly to detect underlying ventricular fibrillation.
Epinephrine or vasopressin should not be administered during CPR as initial drugs unless indicated by an experienced physician.

Finally, the mortality rate after CRA in postoperative cardiac surgery units can be extremely high, ranging from 50 to 70%, according to data from the United States of America. However, this mortality can be significantly reduced to 35% when specific care protocols, such as the Cardiac Surgery Unit Advanced Life Support recommended by the European Association of Cardio Thoracic Surgery and the STS^[5]^, are routinely implemented and used for training and capacity-building in cardiovascular postoperative units.
